# Hospital characteristics and patient populations served by physician owned and non physician owned orthopedic specialty hospitals

**DOI:** 10.1186/1472-6963-7-155

**Published:** 2007-09-25

**Authors:** Peter Cram, Mary S Vaughan-Sarrazin, Gary E Rosenthal

**Affiliations:** 1 Division of General Internal Medicine, Department of Internal Medicine, University of Iowa Carver College of Medicine, Iowa City, IA, USA; 2 Center for Research in the Implementation of Innovative Strategies for Practice (CRIISP), Iowa City Veterans Administration Medical Center, Iowa City, IA, USA

## Abstract

**Background:**

The emergence of physician owned specialty hospitals focusing on high margin procedures has generated significant controversy. Yet, it is unclear whether physician owned specialty hospitals differ significantly from non physician owned specialty hospitals and thus merit the additional scrutiny that has been proposed. Our objective was to assess whether physician owned specialty orthopedic hospitals and non physician owned specialty orthopedic hospitals differ with respect to hospital characteristics and patient populations served.

**Methods:**

We conducted a descriptive study using Medicare data of beneficiaries who underwent total hip replacement (THR) (N = 10,478) and total knee replacement (TKR) (N = 15,312) in 29 physician owned and 8 non physician owned specialty orthopedic hospitals during 1999–2003. We compared hospital characteristics of physician owned and non physician owned specialty hospitals including procedural volumes of major joint replacements (THR and TKR), hospital teaching status, and for profit status. We then compared demographics and prevalence of common comorbid conditions for patients treated in physician owned and non physician owned specialty hospitals. Finally, we examined whether the socio-demographic characteristics of the neighborhoods where physician owned and non physician owned specialty hospitals differed, as measured by zip code level data.

**Results:**

Physician owned specialty hospitals performed fewer major joint replacements on Medicare beneficiaries in 2003 than non physician owed specialty hospitals (64 vs. 678, P < .001), were less likely to be affiliated with a medical school (6% vs. 43%, P = .05), and were more likely to be for profit (94% vs. 28%, P = .001). Patients who underwent major joint replacement in physician owned specialty hospitals were less likely to be black than patients in non physician owned specialty hospitals (2.5% vs. 3.1% for THR, P = .15; 1.8% vs. 6.3% for TKR, P < .001), yet physician owned specialty hospitals were located in neighborhoods with a higher proportion of black residents (8.2% vs. 6.7%, P = .76). Patients in physician owned hospitals had lower rates of most common comorbid conditions including heart failure and obesity (P < .05 for both).

**Conclusion:**

Physician owned specialty orthopedic hospitals differ significantly from non physician owned specialty orthopedic hospitals and may warrant the additional scrutiny policy makers have proposed.

## Background

The emergence of specialty hospitals focusing on narrow procedural aspects of medicine (e.g., cardiology, orthopedics) has generated widespread controversy. One area of controversy has been the allegation by critics that specialty hospitals preferentially admit low-risk patients without demonstrating improvements in risk-adjusted outcomes [1,2]. Recently completed empirical analyses have now addressed this area of concern. Available data suggest that specialty hospitals generally do provide care to lower-risk patient populations than competing general hospitals, but also that specialty hospitals appear to have 10%–20% lower risk-adjusted rates of adverse outcomes [3,4].

Another area of controversy has centered on the precise definition of a specialty hospital and, in particular, the importance of physician ownership of specialty hospitals as a differentiating factor. Critics of specialty hospitals claim that physician ownership of hospitals is extremely concerning because it creates incentives for physicians to take steps to increase utilization of their facilities (so called supplier induced demand), thereby driving up healthcare costs [5,6]. Supporters counter that physician ownership improves quality by better aligning physician and hospital interests [7]. Lost in this debate is a lack of explicit acknowledgement that there are both physician owned specialty hospitals and non physician owned specialty hospitals.

An understanding of potential similarities and differences between physician owned and non physician owned specialty hospitals is of more than academic interest since legislators at both the state and federal levels have responded to the specialty hospital controversy by proposing legislation banning physician ownership of hospitals [8,9]. While such legislation would likely be effective in eliminating the growth in physician owned specialty hospitals, such legislation would do little to prevent continued growth of non physician owned specialty hospitals.

We used Medicare Part A administrative data and American Hospital Association Annual Survey data to answer the following questions: 1) Do physician owned and non physician owned specialty orthopedic hospitals differ with regards to important hospital characteristics including bed size, teaching status, or nurse staffing levels; 2) Do physician owned and non physician owned specialty hospitals differ with respect to the demographic characteristics and comorbidity of the patient populations that they serve; 3) Do physician owned specialty hospitals and non physician owned specialty hospitals differ with respect to the socio-demographic characteristics of the neighborhoods where the hospitals are located.

## Methods

### Data

Consecutive Medicare beneficiaries age 65 years and older who underwent total hip replacement (THR [N = 432,579]) or total knee replacement (TKR [N = 719,482]) between 1999 and 2003 were identified from the Medicare Provider and Analysis Review (MedPAR) Part A public data files using International Classification of Diseases, 9^th ^Clinical Modification (ICD-9-CM) procedure codes (81.51 for primary THR and 81.53 revision THR: 81.54 for primary TKR and 81.55 for revision TKR). The Part A files contain data for Medicare patients discharged from acute care hospitals with the exception of Medicare managed care enrollees. Data elements from the MedPAR data include: patient demographics; primary and secondary diagnoses and procedures, as captured by ICD-9-CM codes; and patients' zip codes of residence. Additional hospital-level information was obtained from the 2003 American Hospital Association Annual Survey, including hospital teaching status, hospital ownership (i.e., for-profit, not for-profit), total number of hospital beds, and hospital registered nurse (RN) staffing. Zip code level socio-demographic information (e.g., per-capita income, median home values) was obtained by linking patients' (and hospital address) zip codes to zip code level data available from the 2000 US Census data [10].

### Definition of specialty orthopedic hospitals and general hospitals

We identified specialty orthopedic hospitals using a methodology that we have used previously, with special care taken to identify whether each specialty hospital had physician owners [3]. Briefly, a measure of orthopedic specialization was created for each hospital defined as the proportion of all 2003 Medicare admissions that were categorized as Major Diagnostic Category (MDC) 8 (Diseases of the Musculoskeletal System). The specialty index was used to identify the 50 hospitals performing major joint replacement (either THR or TKR) with the greatest orthopedic specialization (i.e., highest specialty index ratio). We then excluded from this group all general hospitals, defined as those hospitals that provided obstetric care and/or general pediatric services (N = 10) because it is generally accepted that specialty orthopedic hospitals do not provide either obstetric or pediatric services. This resulted in the identification of 40 specialty orthopedic hospitals whose orthopedic specialization was verified by review of hospital websites and/or confirmatory telephone calls.

We then determined the ownership of each of these 40 specialty hospitals (i.e., physician owned or non physician owned) using two complementary sources of information on specialty hospital ownership. First, we compared our list of specialty orthopedic hospitals with information regarding specialty ownership prepared by the Medicare Payment Advisory Committee (MedPAC) [11,12]. This resulted in the identification of 25 physician owned specialty hospital, 8 non physician owned specialty hospitals; and uncertain ownership for seven specialty hospitals. Second, we compared our list of specialty hospitals with a list of physician owned specialty hospitals published by CMS, resulting in the identification of an additional 4 physician owned specialty hospitals [13]. Thus, we were able to successfully determine the ownership of 37 of 40 specialty orthopedic hospitals, with 29 being physician owned and 8 non physician owned; we were unable to determine the ownership of 3 specialty hospitals and therefore, excluded these hospitals from further analyses.

### Analyses

We compared the characteristics of physician owned and non physician owned specialty hospitals including the number of Medicare admissions in 2003, total number of major joint replacements in 2003, and proportion of admissions falling within MDC8 using the chi-square statistic or Wilcoxon rank-sign test as indicated. Then, we compared the number of hospital beds, proportion of hospitals reporting a formal affiliation with a medical school, and proportion that are for-profit for the physician owned and non physician owned specialty hospitals using similar statistical methods. We compared the nurse staffing ratios for physician owned and non physician owned hospitals as measured by the ratio of RNs employed by each hospital to the total number of hospital beds; this ratio has been used previously as a measure of hospital nursing quality [14].

Next we compared demographic characteristics (e.g., age, sex, race), median per capita income measured at the zip code level, and prevalence of specific comorbid conditions of the populations of patients admitted to physician owned and non physician owned specialty hospitals. Comorbid conditions were determined using algorithms developed by Elixhauser et al [15] as well as additional high-risk clinical conditions specific to joint replacement surgery (prior joint replacement surgery, acute fracture) that have been used in prior analyses assessing orthopedic outcomes [16-18]. Finally, we examined whether physician owned and non physician owned specialty hospitals differed with respect to the socio-demographic characteristics of the neighborhoods where they are located because prior investigations have suggested that hospital location is an important determinant of patients' hospital choice [19,20]. For these analyses we linked the zip code for each hospital's address to zip code level measures available from the census data All p-values are 2-tailed, with p-values ≤ 0.05 deemed statistically significant. All analyses were performed using Stata SE 8.2 (Stata Corp., College Station, TX).

## Results

The 29 physician owned specialty hospitals performed 1,814 THRs and 4,770 TKRs while the 8 non physician owned specialty hospitals performed 8,664 THRs and 10,542 TKRs on Medicare beneficiaries between 1999 and 2003. The 29 physician owned specialty hospitals were located in 10 different states, with five states having three or more (Texas 9, South Dakota 5, and Kansas, Oklahoma, and Washington each having three). The 8 non physician owned specialty hospitals were located in 8 different states. Characteristics of the 29 physician owned specialty hospitals and 8 non physician owned specialty orthopedic hospitals are displayed in Table 1. Physician owned specialty hospitals were significantly smaller than the non physician owned specialty hospitals as indicated by fewer total Medicare admissions in 2003, performance of fewer major joint replacement surgeries, and fewer licensed hospital beds. A significantly larger proportion of physician owned specialty hospitals performed fewer than 50 major joint replacements on Medicare beneficiaries in 2003 (65% [n = 19]) compared to non physician owned hospitals (25% [n = 2]), P = .04). Physician owned specialty hospitals were less likely to be affiliated with a medical school than the non physician owned specialty hospitals (6% vs. 43%, P = .05). Physician owned hospitals were also more likely to be for profit (94%) than non physician owned hospitals (28%). In our examination of nursing staffing ratios, we found that physician owned specialty hospitals averaged 2.2 nurses per licensed bed while non physician owned hospitals had 1.6 nurses per bed, though this difference was not statistically significant (P = .12).

**Table 1:** Characteristics of physician owned and non physician owned specialty hospitals

CHARACTERISTIC	PHYISICAN OWNED (N = 29)	NON PHYSICIAN OWNED (N = 8)	P-VALUE
Mean number of Medicare admissions in 2003, sd	126 (122)	1,305 (1,146)	< .001
Mean number of major joint replacements in 2003, sd	64 (71)	678 (700)	< .001
Mean proportion of admissions in 2003 within MDC8, sd	0.86 (0.14)	0.80 (0.16)	.29
Mean number of hospital beds, (sd)	21 (17)*	73 (39)^†^	< .001
Major teaching hospitals, %	0 (0)*	1 (14)^†^	.11
Medical school affiliation, %	1 (6) *	3 (43)^†^	.05
Number that are for-profit, %	17 (94)*	2 (28)^†^	.001
Mean ratio of FTE nurses to hospital beds, sd	2.2 (.97)*	1.6 (.86)^†^	.12

*Based upon the 18 physician owned specialty hospitals that couldbe matched to the AHA survey

^†^Based upon the 8 non physician owned specialty hospitals that could be matched to the AHA survey

Patients who underwent THR and TKR in physician owned specialty hospitals were slightly younger and more likely to be hispanic than patients in non physician owned specialty hospitals (Table 2); they also lived in less affluent zip codes, as evinced by significantly lower median per capita incomes. Patients treated in physician owned specialty hospitals were less likely to have many common comorbid illnesses than patients treated in non physician owned specialty hospitals. For example, patients in physician owned hospitals had significantly lower rates of diabetes, heart failure, obesity and chronic obstructive pulmonary disease (COPD). In addition, patients treated in physician owned specialty hospitals had fewer high-risk orthopedic conditions including previous joint replacement surgery and fracture at the time of surgery.

**Table 2:** Demographics, socioeconomic measures, and comorbidity of patients who underwent THR and TKR in physician owned and non physician owned specialty hospitals

	**THR**			**TKR**		
Characteristic	MD Owned n = 1,814	Non MD Owned n = 8,664	P-value	MD Owned n = 4,770	Non MD Owned n = 10,542	P-value

DEMOGRAPHICS						
Age, mean (SD), (years)	75.1 (6.4)	75.4 (6.3)	.02	75.0 (6.2)	75.3 (6.2)	< .001
Sex, women, %	1,137 (62.7)	5,367 (62.0)	.36	3,113 (65.3)	6,736 (63.9)	.10
Race						
Non-Hispanic white, %	1,694 (93.4)	8,233 (95.1)	.005	4,270 (89.5)	9,621 (91.3)	.001
Black, %	46 (2.5)	276 (3.1)	.145	89 (1.8)	668 (6.3)	< .001
Hispanic, %	36 (2.0)	23 (0.3)	< .001	268 (5.6)	59 (0.5)	< .001
Asian, %	*	20 (0.2)	.93	*	32 (0.3)	.13
Median per-capita income ($), (sd)	21,800 (10,480)	32,450 (19,500)	< .001	19,800 (9,000)	28,300 (16,600)	< .001
COMORBIDITY						
Diabetes						
All, %	134 (7.3)	749 (8.7)	.08	517 (10.8)	1,276 (12.1)	.03
With complications, %	*	35 (0.4)	.06	*	75 (0.7)	< .001
Congestive heart failure,%	*	276 (3.2)	< .001	34 (0.7)	340 (3.2)	< .001
Renal failure, %	0 (0.0)	26 (0.3)	.02	*	48 (0.4)	.001
Obesity, %	50 (2.8)	472 (5.5)	< .001	186 (3.9)	973 (9.2)	< .001
COPD, %	97 (5.3)	1,012 (11.7)	< .001	299 (6.3)	1,207 (11.5)	< .001
Previous Joint Replacement, %	210 (11.6)	1,839 (21.2)	< .001	211 (4.4)	809 (7.7)	< .001
Fracture, %	17 (0.9)	225 (2.6)	< .001	n/a	n/a	
Admitted as transfer from another acute care hospital, %	*	45 (0.5)	.007	*	*	.71

* numbers of patients in these cells were small (< = 12). These numbers were suppressed to protect the confidentiality of patient information in accordance with Medicare policies.

In our evaluation of hospital location, the majority of both the physician owned (90% [n = 26]) and non physician owned (75% [n = 6]) specialty hospitals were located in urban areas as measured by a rural-urban commuting area of 1 [21]. We also found that physician owned specialty hospitals were located in zip codes with lower household income, a higher proportion of residents on public assistance, a higher proportion of black residents and a lower proportion of white residents as compared with non physician owned specialty hospitals, though these differences were not statistically significant (Table 3).

**Table 3:** Socio-demographic measures for physician owned and non physician owned specialty hospitals

CHARACTERISTIC	PHYISICAN OWNED (N = 29)	NON PHYSICIAN OWNED (N = 8)	P-VALUE
Median per-capita income ($), (sd)	26,900 (15,200)	35,300 (27,900)	.26
Percent of population on public assistance, (sd)	2.7 (2.8)	2.3 (1.9)	.66
Percent white, (sd)	70.1 (25.5)	76.8 (24.5)	.54
Percent black, (sd)	8.2 (12.8)	6.7 (10.3)	.76

## Discussion

Our findings suggest that physician owned and non physician owned specialty orthopedic hospitals differ significantly both in terms of hospital characteristics and the patient populations they serve. Physician owned hospitals tend to be smaller than non physician owned hospitals, are less likely to be affiliated with medical schools, and are more likely to be for-profit. In addition, physician owned specialty hospitals admit Medicare patients who are slightly younger and have fewer comorbid conditions than non physician owned specialty hospitals. Finally, physician owned specialty hospitals are located in zip codes that are less affluent and have a higher proportion of black residents than non physician owned specialty hospitals, though these differences were not statistically significant.

A number of our findings are important and merit further comment. Prior studies have demonstrated that specialty hospitals tend to be smaller and less likely to have academic affiliations than general hospitals [5,22]. Likewise, prior studies have found that specialty hospitals admit patients with less comorbid illness than competing general hospitals [3,4]. However, these studies have been inconsistent with regards to their definitions of specialty hospitals. In particular, investigators and policy makers have failed to clearly define whether specialty hospitals should be identified broadly as any hospital that specializes on a particular group of diseases or conditions, or should be defined more narrowly as specialized hospitals with physician ownership. Our findings that both the characteristics of physician owned specialty hospitals and the populations of patients served by these hospitals differ from non physician owned specialty hospitals suggests that physician owned specialty hospitals are distinct.

The findings with regard to hospital location are also interesting. Given the prominent role that hospital location could be expected to play in hospital choice, it would be reasonable to expect that the socio-demographic characteristics of the patients admitted to physician owned and non physician owned specialty hospitals would reflect the socio-demographics of the neighborhoods in which these hospitals are located. Thus, our finding that physician owned specialty hospitals admit less affluent patients as measured by the patients' zip codes of residence makes sense because the physician owned specialty hospitals are located in less affluent neighborhoods than the non physician owned hospitals. Conversely, our finding that physician owned specialty hospitals admit a smaller proportion of black patients than non physician owned specialty hospitals despite being located in zip codes with a greater proportion of black residents is somewhat difficult to explain. One possibility is that black patients are choosing not to go to physician owned specialty hospitals, while another is that the physicians who treat black patients are consciously referring these patients to other facilities. Alternatively, it is well documented that blacks have shorter life expectancies than white patients; thus, many blacks may actually die before reaching the age of Medicare eligibility (i.e., age 65), and thereby depressing the proportion of black patients in the Medicare population [23].

These findings have a number of important implications for the ongoing policy debate over specialty hospitals. At the most basic level, our findings suggest that legislative efforts to ban physician investment in specialty hospitals [24] are likely to be effective in limiting the growth of the sub-group of specialty hospitals that most obviously engage in providing care to low-risk patient populations- physician owned specialty hospitals. While such legislation is likely to be effective, it is also likely to have a number of repercussions that warrant careful consideration.

First, banning physician investment in specialty hospitals would have the potential to stifle benefits that may result from physician ownership of hospitals. Potential benefits that have been cited include improved patient outcomes, improved hospital efficiency and cost savings, and enhanced patient satisfaction; some of these purported benefits are substantiated by published data, while others remain hypothetical [7]. For example, a growing body of literature now suggests that as a group specialty hospitals offer a 10%–20% improvement in patient outcomes when compared to general hospitals [3,25]. While some of the improved outcomes in specialty hospitals is certainly related to the fact that specialty hospitals provide care to a healthier cohort of patients, analyses have demonstrated improved outcomes in specialty hospitals persist after adjustment for patient characteristics [3,25]. While there remains concern that some of the improved outcomes observed in specialty hospitals may be an artifact on unmeasured differences in patient severity, in aggregate it increasingly appears as if specialty hospitals may deliver improved patient outcomes. Studies assessing efficiency and costs are much more limited and less clear as to the benefits specialty hospitals afford. A recent study by Barro et al. suggest that specialty hospital entry into healthcare markets may serve to restrain growth in healthcare costs [26,27]. Alternatively, Nallamothu et al. found evidence that specialty hospitals may actually increase costs by driving increased utilization of cardiac revascularization [5]. Studies have failed to demonstrate that specialty hospitals reduce patient hospital length of stay- a potential measure of hospital efficiency [3]. Rigorous empirical data comparing the satisfaction of patients treated in specialty and general hospitals are not currently available. Thus specialty hospitals appear to offer improvements in patient outcomes, but their impact on medical costs and patient satisfaction are currently unknown. In addition, it is unclear how much of these benefits, if any, are due to physician ownership versus hospital specialization and how these benefits would be affected if physician ownership was prohibited.

Second, while a ban on physician investment in hospitals would be likely to curtail the growth in physician owned specialty hospitals, it would do little to slow the growth in non physician owned specialty hospitals. Across the United States hospitals faced with stagnant reimbursement and growing numbers of uninsured and under-insured patients are expanding their efforts to grow profitable service lines [28]. In turn, general hospitals are increasingly developing ambulatory surgery centers, free standing specialty hospitals, and autonomous "hospitals-within-a-hospital" in an effort to grow profits; banning physician investment in specialty hospitals is unlikely to have a significant impact on this trend. Third, a ban on physician owned specialty hospitals, in the absence of reform in the prospective payment system is unlikely to curtail the growth in niche hospitals developed to take advantage of well recognized inconsistencies in the current reimbursement system [29].

This study has a number of important limitations that should be discussed. First, our assessment of physician ownership of specialty hospitals dichotomized specialty hospitals into physician owned and non physician owned groups. In actuality, it is well recognized that the financial relationships between physicians and specialty hospitals are highly variable [12]. Some hospitals have a limited number of physician owners, each of whom own a significant fraction of the hospital while other hospitals have many physician owners, each of whom has a minimal investment in the facility. These differences have the potential to significantly impact the behavior of physician owners. Second, this study was limited to analyses of Medicare beneficiaries and thus extending our findings to other patient populations should be done with care. Third, this analysis was limited to orthopedic specialty hospitals. While orthopedic specialty hospitals represent the single largest category of specialty hospitals, it is unclear whether they can be generalized to other categories of specialty hospitals [30]. Fourth, the number of hospitals included in this analysis was small. This small sample size limited our statistical power and makes it difficult to establish, with confidence whether differences in hospital characteristics such as nurse staffing ratios or hospital location are significant. Fifth, because this study relied upon Medicare Part A data, we were unable to examine the number of physicians who performed procedures at each hospital or their corresponding procedural volumes.

## Conclusion

In summary, the current study provides evidence that physician owned specialty hospitals differ significantly from non physician owned specialty hospitals, both in terms of hospital characteristics and in terms of the patient populations they serve. Banning physician investment in "whole hospitals" is likely to be effective in limiting growth in the largest subgroup of specialty hospitals, but also has the potential to limit innovation in the hospital delivery system.

## Competing interests

The author(s) declare that they have no competing interests.

## Authors' contributions

PC had full access to all the data in the study and takes responsibility for the integrity of the data and the accuracy of the data analysis. All authors contributed equally to this analysis and approved the final manuscript.

## Pre-publication history

The pre-publication history for this paper can be accessed here:


